# Dermatofibrosarcoma protuberans: from translocation to targeted therapy

**DOI:** 10.7497/j.issn.2095-3941.2015.0067

**Published:** 2015-12

**Authors:** Jonathan Noujaim, Khin Thway, Cyril Fisher, Robin L. Jones

**Affiliations:** Sarcoma Unit, Royal Marsden NHS Foundation Trust, London SW3 6JJ, UK

**Keywords:** Dermatofibrosarcoma protuberans (DFSP), imatinib, Mohs micrographic surgery (MMS), translocation, targeted therapy

## Abstract

Dermatofibrosarcoma protuberans (DFSP), the most common dermal sarcoma, is a low-grade, slow growing fibroblastic malignant neoplasm that most frequently affects middle aged adults and is characterized by a high local recurrence rate and a low propensity for metastasis. Wide surgical resection or Mohs micrographic surgery (MMS) are the preferred approaches for localized disease, while radiation therapy is warranted for inoperable disease or for cases with positive margins where re-excision is not possible. DFSP is generally regarded as refractory to conventional chemotherapy. Treatment options for systemic disease were limited until the discovery of a unique translocation, t(17;22)(q22;q13) (*COL1A1*;*PDGFB*) found in a majority of cases. In recent years, imatinib, a PDGFβR, ABL and KIT inhibitor, has revolutionized systemic therapy in DFSP. In this review, we summarize the epidemiological, clinical, histological and genetic characteristics of DFSP and update the readers on its current management.

## Introduction

Dermatofibrosarcoma protuberans (DFSP), although rare, is one of the most common dermal sarcomas^1^. It is a low-grade, slow growing fibroblastic malignant neoplasm most frequently affecting middle aged adults and characterized by a high local recurrence rate and a low propensity for metastasis. Clinically, DFSP consists initially of a slow growing violaceous, blue-red or brown indurated plaque confined to the dermis with subsequent nodules appearing at later stages. Histologically, it is represented by neoplastic spindle cells arranged in storiform or cartwheel patterns, diffusely infiltrating the dermis and subcutis. Nuclei generally show no atypia and the mitotic count is low. On immunohistochemical (IHC) studies, tumors typically show diffuse expression of CD34. Despite surgical resection, a substantial number of patients will recur and an unfortunate few will eventually develop metastatic disease. Compared to other sarcoma subtypes, DFSP is generally regarded as refractory to standard chemotherapy. A breakthrough in systemic treatment was made possible following the identification of a unique translocation t(17;22)(q22;q13) (*COL1A1*;*PDGFB*) in a majority of cases. This discovery subsequently led to the evaluation of imatinib, a PDGFβR, ABL and KIT inhibitor in the treatment of DFSP. In this review, we summarize the epidemiological, clinical, histological and genetic characteristics of DFSP and update the readers on its current management.

## Epidemiology and clinical features

**Figure 1:**
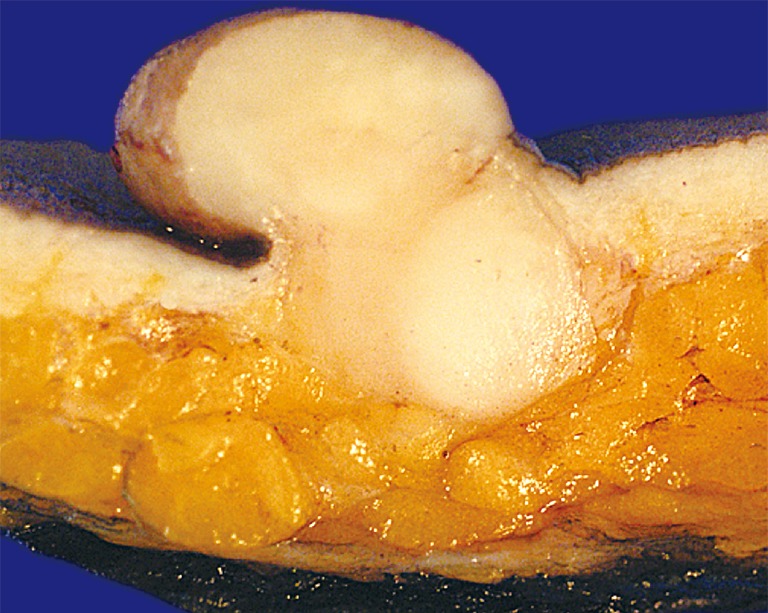
A large polypoid nodule is presented with a homogeneous firm yellowish white cut surface. The tumor can be seen to expand the entire dermis, and extends into the more superficial subcutis.

The incidence of DFSP is estimated to be around 0.8-4.2 cases per million persons per year^2^. Due to better recognition and improvement in ancillary diagnostic modalities, the number of reported cases has increased steadily over the last decades. While DFSP most frequently affects middle age adults, it has been reported in adolescents as well as adults up to the 9th decade (age ranged 15 to over 90 years). There is a slight male predominance^3^^,^^4^. Pediatric cases of DFSP account for 6% of all diagnoses and require a high degree of suspicion^5^. A recent review of the literature shows that several pediatric cases have been reported so far; this might suggest that the number of infants with the condition might be larger than that estimated previously^6^. Clinical presentation of DFSP can be so diverse, particularly in children and adolescence that the differential diagnosis may not lead to malignancy. A biopsy is often the first step that can help to find the tumor as early as possible. Clearly, therefore, an accurate knowledge of the disease is the prerequisite for a wider recognition and appropriate treatment. The most commonly affected anatomical sites are the trunk (40%-50%), the proximal extremities (30%-40%), and the head and neck (10%-15%)^3^^,^^7^^,^^8^. Patients generally report a long history, ranging from months to years, of a slow growing indurated plaque. Lesions have a hard consistency and are fixed to the skin and may appear violaceous, blue-red or brown. They can vary considerably in size, ranging from 2 cm at early stages to 30 cm and larger in neglected cases, although size has not been shown to correlate with prognosis^8^^,^^9^. The disease usually remains contained in the dermis and subcutis, although recurrent or long standing cases may invade deeper structures including the fascia, muscle, periosteum (particularly in cases affecting the scalp), and bone. At an early (‘preprotuberant’) stage, DFSP can be classified into three different forms: a morphea-like form mimicking a scar, morphea, morphea-form basal cell carcinoma, or a dermatofibroma plaque; an atrophoderma-like form similar to atrophoderma or anetoderma; and finally an angioma-like form resembling vascular malformations^10^. Eventually, one or multiple nodules may appear in the protuberant phase (**Figure 1**). Efforts should be made in excluding fibrosarcomatous transformation, which may occur *de novo* or upon recurrence, in patients reporting rapid enlargement of their neoplasm^11^^-^^13^. While the development of metastatic disease is rare (5%), rates of local recurrence are significant (20%-50%)^7^^,^^8^^,^^14^, with median time to local recurrence of around 32 months; long-term follow-up is therefore mandatory^3^. The lungs are the primary site for distal spread; although nodal involvement is reported uncommon^4^. The global prognosis of DFSP is excellent, with reported 2- and 5-year survival rates of 97% and 92%, respectively^15^.

## Histology

**Figure 2:**
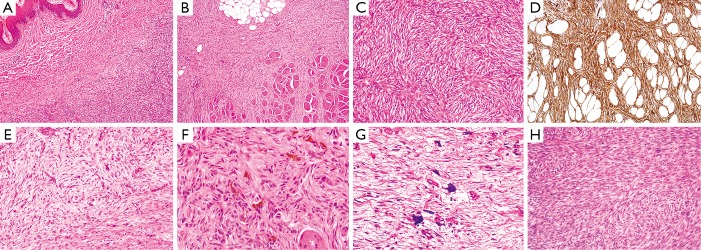
(A) Dermatofibrosarcoma protuberans (DFSP). Ill-defined cellular tumor is present within the superficial dermis, close to the squamous epithelium (top left of field) (H&E staining, 20×). (B) Most DFSP infiltrates the dermis and subcutis, but some are deeply infiltrating. This example shows prominent infiltration of skeletal muscle bundles (lower half of field) (H&E staining, 20×). (C) DFSP typically comprises cellular distributions of bland spindle cells with elongated vesicular nuclei and small amounts of fibrillary cytoplasm, in a prominent storiform or ¸cartwheel̓ pattern (H&E staining, 200×). (D) Immunohistochemically, DFSP characteristically shows diffuse and strong expression of CD34. This example also illustrates the linearly oriented tumor strands that infiltrate the subcutaneous fat in a ¸honeycomb̓ pattern (IHC staining, 100×). (E) Tumors may show focal or sometimes prominent myxoid change. As the characteristic storiform architecture is lost, there may be difficulty in establishing a diagnosis of DFSP (H&E staining, 100×). (F) Bednar tumor is characterized by spindle cells in a storiform pattern in which there are scattered pigmented, dendritic melanocytic cells (H&E staining, 400×). (G) In children, giant cell fibroblastoma (GCF) is considered a variant of DFSP, but GCF are typically hypocellular, with bland spindle cells and interspersed tumor giant cells in patternless distributions within myxoid or collagenous stroma (H&E staining, 400×). (H) In fibrosarcomatous transformation, the cells are present in more loosely fascicular distributions or ¸herringbone̓ patterns, and often show more prominent mitotic activity (H&E staining, 100×).

On histopathological examination, DFSP is usually centered in the dermis and subcutis, and is an ill-defined, cellular neoplasm composed of storiform or ‘cartwheel’ distributions of bland, relatively monotonous spindle cells, with elongated nuclei with even chromatin, minimal cytological atypia and small amounts of fibrillary cytoplasm, within collagenous stroma (**Figure 2A-C**). The mitotic count is usually low. The tumor often infiltrates the subcutis in characteristic, linearly orientated strands that show a ‘honeycomb’ pattern. Immunohistochemically, tumors show diffuse and strong expression of CD34 (**Figure 2D**)^16^, but are negative for other markers such as cytokeratins, desmin, smooth muscle actin (SMA) and S100 protein. CD34 positivity supports a diagnosis of DFSP, but is not specific as its expression is also found in a variety of spindle cell neoplasms in the histological differential diagnosis of DFSP, including some benign fibrohistiocytic lesions, solitary fibrous tumor and some undifferentiated spindle cell sarcomas. Furthermore, CD34 expression is also seen in a variety of neoplasms of other lineages, including vascular and hematopoietic tumors. Apolipoprotein D is also strongly expressed in DFSP and may be helpful in supporting its diagnosis and differentiating it from fibrous histiocytoma^17^.

While morphologically conventional DFSP accounts for more than 90% of cases, a number of histologic subtypes have been described. On occasions, there can be prominent myxoid stroma (myxoid DFSP) (**Figure 2E**)^18^^,^^19^, or rarely myoid or myofibroblastic differentiation, in which there are interspersed bundles of bland and SMA-positive myofibroblastic cells. Pigmented DFSP (also known as Bednar tumor) (**Figure 2F**)^20^^,^^21^ is characterized by spindle cells in a storiform pattern in which there are scattered pigmented, dendritic melanocytic cells. It is more commonly found in black patients and behaves similarly to conventional DFSP. Atrophic DFSP is identified by a depressed plaque with a reduced dermal thickness thereby displacing the subcutis closer to the epithelium^22^^,^^23^. Sclerosing DFSP shows less cellular foci, with homogenous collagen bundles, although other areas are morphologically identical to conventional DFSP^24^^-^^26^. Granular cell variant of DFSP is rarely reported and consists of spindle cells mixed with a population of cells with abundant lysosomal granules, round eccentric nuclei, and prominent nucleoli^27^^,^^28^. In children, giant cell fibroblastoma (GCF) is considered a variant of DFSP and shares a common morphological, clinical and genetic background^29^^,^^30^. Areas of DFSP are seen in approximately 15% of GCF^29^. Areas of GCF are typically hypocellular, with bland spindle cells and interspersed tumor giant cells in patternless distributions within myxoid or collagenous stroma (**Figure 2G**). Finally, tumors that have undergone fibrosarcomatous transformation show a more loosely fascicular architecture (**Figure 2H**), rarely with cellular atypia, as well as more prominent mitotic figures, and often show loss of CD34 expression^11^^,^^31^. These changes are found in 10%-20% of cases, and comprise the most aggressive variant, associated with the highest risk of local recurrence and distant metastasis, and overall, are associated with a worse prognosis^32^^,^^33^.

## Genetic background

Early cytogenetic studies initially documented the presence of supernumerary ring chromosomes in DFSP and subsequent fluorescence *in situ* hybridization (FISH) studies and comparative genomic hybridization (CGH) concluded that these rings derived from the centromere of chromosome 22 and interspersed sequences of chromosome 17^34^^-^^37^. Groundbreaking molecular work further revealed that these rearranged chromosomes contained sequences of α1 type I collagen preprotein gene (*COL1A1*;17q21) and platelet-derived growth factor β-chain gene (*PDGFB*;22q13)^38^. In approximately 90% of cases (and regardless of histological pattern), tumors have the characteristic reciprocal translocation t(17;22)(q22;q13), with a majority in the form of supernumerary ring chromosomes. The *COL1A1* breakpoint is highly variable (spanning the exon 6 to exon 49 region) compared to the *PDGFB* breakpoint consistently found in intron 1. The resultant gene fusion leads to constitutive upregulation of *PDGFβ* expression^39^. PDGF, a potent mitogen for mesenchymal cells, is involved in major signaling pathways including Ras-MAPK and PI3K-AKT-mTOR, and in stimulating cell growth, differentiation, and migration^40^^,^^41^. The fusion protein COL1A1-PDGFβ is processed to a mature PDGF-BB homodimer and activates the PDGFβ receptor (PDGFβR) leading to autocrine activation and subsequent tumorigenesis. It is highly probable that the acquisition of t(17;22)(q22;q13) is an early event in the clonal evolution of the disease because of the pre-clinical evidence demonstrating that transfection of the gene rearrangement is capable of transforming normal cells to neoplastic cells^42^^,^^43^.

The characteristic t(17;22) of DFSP can be detected in routine practice using FISH or multiplex reverse transcription polymerase chain reaction (RT-PCR) studies. FISH studies are easy, sensitive and only require *PDGFB* or *COL1A1-PDGFB* fusion probes whereas multiplex RT-PCR requires multiple *COL1A1* primers because of the hypervariable breakpoint region^44^^-^^46^. The t(17;22) translocation is also identifiable in DFSP variants including pigmented DFSP, myxoid DFSP and fibrosarcomatous DFSP^19^. Fibrosarcomatous transformation can present *de novo* or result from long standing disease. Interestingly, genomic gains of *COL1A1-PDGFβ* were found in areas where DFSP evolved into fibrosarcomatous DFSP, once again providing evidence of its oncogenic properties^47^. However, other genomic events including microsatellite instability and *p53* mutations are also involved in tumor progression in DFSP^48^. Beyond the oncological properties of the fusion gene *COL1A1-PDGFβ*, little is known about the other mechanisms contributing to its tumorigenicity. The overexpression of p-STAT3, p-ERK and thrombospondin-1 were highlighted in IHC studies and may possibly contribute to its oncogenesis^49^^,^^50^. Activation of epidermal growth factor receptor (EGFR) and insulin receptor were also noted in a majority of clinical samples^51^. Furthermore, the role of miRNAs remains unexplored, but may play a pivotal role. Recently, down-regulation of miR-205 in DFSP was shown to lead to an overexpression of LRP-1 and possibly ERK phosphorylation thereby contributing to cellular proliferation^52^. Little is known about the mechanisms involved in *COL1A1-PDGFβ* negative DFSP. A case report described a DFSP patient lacking the characteristic *COL1A1-PDGFβ*, but instead presented a complex translocation between chromosomes 5 and 8, involving the *CSPG2* gene at 5q14.3 and the *PTK2B* gene at 8p21.2^53^. Clearly, more work is needed to fully understand the molecular background of DFSP, with a particular attention to t(17;22) negative DFSP.

## Treatment

The current treatment options of DFSP were reviewed systematically by Rutkowski *et al.*^54^. In the following sections, we will highlight the important aspects on its management.

### Surgery

The mainstay of management of localized disease is complete surgical resection^7^. Because of the locally invasive nature of DFSP, obtaining clear surgical margins with particular attention to the deep fascia is very important. When DFSP was initially approached with conservative margins, the reported recurrence rates were up to 60%^4^. By further removing the tumor *en bloc* with 2-5 cm margins, recurrence rates dropped around 6% but remained significant^14^^,^^55^^-^^57^. The underlying reason why DFSP tends to relapse despite wide surgical excision is explained by its tendency to invade outwards from the original focus of disease. At the time of surgery, these microscopic projections are not observable^8^. By further widening the excision in order to obtain more than 5 cm clear margins, the recurrence rates drop to almost 0%^58^^,^^59^. However, this comes at a cost of an increased risk of complication as well a sub-optimal cosmetic result. In recent years, outcomes following surgical resection were improved by the use of Mohs micrographic surgery (MMS). MMS is a technique that involves immediate examination of the microscopic margin^60^^-^^64^. The technique is repeated several times until the process is complete. This of course can enhance the cosmetic result and is important to consider in children and young adults. In addition, it can be particularly useful in lesions at challenging anatomic sites, where performing radical surgery can be difficult^57^^,^^65^^,^^66^. Lateral safety margins of 1-1.3 cm are sufficient^67^. Although some reviews suggest lower recurrence rates (around 1%) with MMS compared to conventional wide surgical resection (6.3%)^56^^,^^57^^,^^62^^,^^63^^,^^65^, conclusions cannot be drawn because of a lack of randomized trials. Despite this shortcoming, when available, MMS should be the privileged approach^67^. If unavailable, wide surgical resection with 3 cm margins is sufficient^67^^,^^68^.

### Radiation therapy

There is no indication of adjuvant radiation therapy following an R0 resection. It may have a role following resection of a tumor with close or positive margins in which further surgery is not possible, especially when fibrosarcomatous transformation has occurred^69^. In addition, radiation may be employed as primary therapy in patients with unresectable disease^70^^-^^72^. The target volume should include the primary tumor volume in addition to a safety margin of 3-5 cm. The total recommended dose varies from 60 Gy for microscopic disease to 70 Gy for macroscopic disease in 2 Gy fractions^67^.

### Imatinib therapy

An unfortunate minority of patients will subsequently metastasize during the course of their disease. DFSP is generally perceived as being resistant to conventional chemotherapy^71^^,^^73^. There is some anecdotal evidence of response to methotrexate, although the experience is limited^74^^,^^75^. A breakthrough in the management of metastatic patients was possible following the discovery of the translocation t(17;22)(q22;q13) (*COL1A1*;*PDGFB*), whereby efforts were directed towards targeting PDGFR. Imatinib is an inhibitor of PDGFβR, ABL and KIT, and blocks PDGF signaling, interfering with phosphorylation of the receptor tyrosine kinase. Pre-clinical studies showed that imatinib had *in vitro* and *in vivo* activity via the induction of apoptosis (rather than reducing proliferation)^76^^,^^77^. Imatinib activity was also observed in tumors expressing low levels of PGDFRB. Recent transcriptional profiling of imatinib-treated tumor samples was suggestive of a possible enhanced susceptibility of IM-treated tumor cells to an antigen specific, HLA-restricted immune recognition^78^. In 2002, two clinical reports documented the activity of this drug in patients with inoperable or metastatic disease^79^^,^^80^. A patient with locally advanced DFSP achieved a response to imatinib enabling sufficient downstaging for surgery to be performed. On histopathological examination, there was a pathological complete response. Another patient, with DFSP with fibrosarcomatous change, did not respond to imatinib^79^. The other report documented a good response in a patient with a tumor harbouring the t(17;22) translocation^80^. The clinical activity of imatinib was subsequently described in several other case reports^81^^,^^82^.

The Imatinib Target Exploration Consortium Study B2225 proceeded to show that eight patients with locally advanced disease harboring the t(17;22) translocation responded to 800 mg of imatinib (4 obtaining complete responses). No response was documented in a metastatic patient lacking the translocation^83^. The results of this study subsequently led to Food and Drug Administration (FDA) approval of imatinib for DFSP. Similar results were also observed for DFSP patients enrolled in a phase II trial studying imatinib in tumors with imatinib sensitive tyrosine kinases^84^. A combined analysis of the SWOG and EORTC phase II trials (prematurely closed because of slow recruitment) in patients with locally advanced and metastatic DFSP has been published^85^. In the EORTC trial patients were required to have the PDGFβ rearrangement to be eligible to enter the trial, and patients could undergo surgery at 14 weeks if this was deemed feasible. Patients entered into the EORTC trial (*n*=16) were treated with imatinib 400 mg twice per day while those in the SWOG trial (*n*=8) received 400 mg once per day. A total of 24 patients were included in the analysis with a median follow-up of 2.6 years; 11 (46%) achieved a Response Evaluation Criteria in Solid Tumors (RECIST) partial response and four experienced progressive disease. The median time to progression was 1.7 years. This trial confirmed the previous reported activity of imatinib in DFSP, but also suggested that 400 mg once a day may be sufficient.

### Neo-adjuvant imatinib

Imatinib was also studied in a neo-adjuvant setting with the aim to improve on surgical outcomes. A multicenter phase II trial of neoadjuvant imatinib 600 mg once a day for a 2-month period prior to surgical resection reported a clinical response rate in 9 of 25 patients (36%)^86^; the lower response rate here can be explained by the short duration of imatinib treatment. A further multicenter phase II trial evaluated neoadjuvant imatinib 600 mg once a day for a minimum of 6 weeks^51^. Thereafter if the patient had at least stable disease, imatinib was continued at the discretion of the investigator until the planned surgery. The reported response rate was 57.1%, similar to previous imatinib trials. The median treatment duration was 3.1 months and the median follow-up was 6.4 years. Only one patient developed secondary resistance to imatinib. Follow-up did not support smaller surgical margins following successful response to imatinib. These studies show once again that lower doses of imatinib are sufficient and well tolerated in treatment of DFSP. However, it is difficult to comment on the impact of neo-adjuvant imatinib therapy on surgical margins and recurrence rates. The initial French study proceeded with wide excision but did not document any follow-up^86^ while the Ugurel *et al.*^51^ trial proceeded to narrow to intermediate excision (<2 cm). Therefore, direct comparison is impossible. The routine use of neo-adjuvant imatinib for operable DFSP cannot be recommended and should remain investigational. However, its use is warranted in borderline resectable cases. Under these circumstances, neo-adjuvant imatinib should be continued until a maximal response is documented before proceeding to surgery.

### Imatinib resistance and translocation negative DFSP

Patients lacking the t(17;22) translocation do not usually respond to imatinib^51^^,^^83^^,^^86^. Furthermore, the pigmented-variant DFSP seems to be unresponsive to imatinib despite cases presenting the translocation, although larger cohorts will be needed to validate these findings^51^^,^^85^. To our knowledge, there are currently no trials investigating novel agents designed specifically for DFSP. Treatment options available for translocation negative or imatinib-resistant DFSP are limited. Single case reports have suggested that other tyrosine kinase inhibitors such as sunitinib^51^ and sorafenib^73^^,^^87^ may have some activity in tumors resistant to imatinib. Further investigations are required to evaluate the full spectrum of activity of these drugs. DFSP with fibrosarcomatous transformation can respond to imatinib; however, resistance appears rapidly^78^^,^^88^^,^^89^. Because fibrosarcomatous transformation carries a worse prognosis, patients with inoperable or metastatic disease should be treated with conventional anthracycline-based schedules following imatinib failure^90^.

Efforts are now directed towards understanding imatinib resistance in DFSP. Whole genome sequencing in a patient with DFSP who subsequently developed imatinib resistance did not reveal point mutations in the *PDGFB* gene^91^. Instead, novel mutations were found in genes implicated in various signaling pathways including NK-κB. Therefore, it is possible that resistance is secondary to other mechanisms rather than the acquisition of new mutations in the *COL1A1-PDGFB* fusion gene. Recently, homozygous deletion of *CDKN2A* and *CDKN2B* were identified in pre-clinical imatinib-resistant models^92^. CDK4/6 inhibitors were able to inhibit cellular proliferation *in vitro* and warrant further investigation.

## Conclusion

DFSP is a rare soft tissue sarcoma subtype with an infiltrative growth pattern and low rate of metastatic disease. The mainstay of management of localized disease is surgical resection, and MMS or wide surgical resection is the current accepted approaches. Radiation can be administered following resection with close or positive margins (if re-excision is impossible) and can be primary therapy for patients with inoperable disease. Conventional chemotherapy has limited activity in this disease. In the subset of patients with inoperable or metastatic disease, imatinib therapy is warranted following confirmation of t(17;22)(q22;q13) (*COL1A1*;*PDGFB*). A starting dose of 400 mg once a day is sufficient and better tolerated. For imatinib-resistant patients, options are limited and include sorafenib and sunitinib. Enrollment in clinical trials is strongly encouraged. Efforts should now be directed towards better understanding the biology of DFSP in order to identify novel targets.
